# Editorial: International Day of Happiness 2023: caring for ourselves so that we may care for others

**DOI:** 10.3389/fpubh.2024.1527659

**Published:** 2024-11-27

**Authors:** Andrew T. Olagunju, Jutta Lindert

**Affiliations:** ^1^Department of Psychiatry and Behavioral Neurosciences, McMaster University, Hamilton, ON, Canada; ^2^Forensic Psychiatry Program, St. Joseph's Healthcare Hamilton, Hamilton, ON, Canada; ^3^Federal Neuropsychiatric Hospital, Calabar, Cross River State, Nigeria; ^4^Discipline of Psychiatry, University of Adelaide, Adelaide, SA, Australia; ^5^Department of Psychiatry and Behavioral Sciences, University of Oklahoma, Oklahoma City, OK, United States; ^6^Department of Social Work and Health, University of Applied Sciences Emden/Leer, Emden, Germany

**Keywords:** caregiving, happiness & wellbeing, global health, kindness & caring, self-care, mental health, collective wellbeing

The 2023 International Day of Happiness was earmarked by the United Nations (UN) with a special theme—Be Mindful, Grateful and Kind. This theme is unique for several reasons. Most importantly, it echoes the interconnectedness of everyone's health and underscores the importance of everyone to support the call to foster collective wellbeing-happiness, knowing that an individual's health is the health of others and vice versa (1). In no time is caring for ourselves and others more pressing than now, especially as the world emerges from the pandemic, and grapples with the global ramifications of diverse health emergencies from conflicts, disasters, and climate change among others (2, 3).

The significance of happiness to our collective wellbeing and mental health, characterized by a state of positive emotions, contentment, prosperity, and meaningful living for everyone cannot be over-emphasized. However, this cannot be attained without universal support for regular use of caring for others and for one self (e.g., reducing unkindness, discrimination, and bullying) to promote emotional well-being and happiness around the world (4, 5). Attaining a positive emotional state can help individuals, families and communities.

In support of the spirit behind the 2023 UN International Day of Happiness, and recognizing the significance of collective wellbeing and happiness, this Research Topic was launched to publish a collection of new articles, highlighting trends across diversified settings on how caregivers are promoting personal wellbeing, and communication to help them provide optimal care to patients and their loved ones. In particular, the articles in this collection address the wellbeing of formal and informal caregivers, describing the beneficial effects of positive mental health activities or interventions (including mindfulness, self-care, resilience building, non-violent communication, storytelling, and mental health advocacy) across international settings and populations.

Redublo et al. present findings from participatory action research using a novel online workshop series to explore the personal recovery journeys of family caregivers of a sample of individuals with mental illness in the province of Ontario, Canada. Seven recovery-oriented topics (including self-care, resilience building, non-violent communication, storytelling, and mental health advocacy), were identified as important for the personal recovery of family caregivers and their ability to provide optimal care for their loved ones. Among a sample of nurses in three provinces in China, Li et al. reported the potential benefits of higher educational-professional attainment, perceived social support, mindfulness, and self-care to confer a higher level of resilience on nursing staff. Through a qualitative interview of caregivers and hospice staff in Scotland and England during the COVID-19 pandemic, Bailey et al. shed more light on the decision-making behind the place of end-of-life care and the impact it had on the experiences of end-of-life care during the global pandemic. In a study conducted among Health Care Providers' in the Indian State of Kerala, Joseph et al. highlighted the importance of informational management to the continuity of care of patients and identified better knowledge, skills, professional role, identity, beliefs about capabilities, intentions, goals, and optimism as crucial enablers for continuity of care. Lastly, Dysart and Harden highlighted the important role of workplace wellness intervention that involves physical activity, mindfulness, and self-care to better performance and personal improvement among extension workers and leaders in the Commonwealth of Virginia in the United States.

In sum, the articles published in this Research Topic highlight the crucial role of positive mental health, selfcare, and being kind to each other, mental health promoting attitudes and communication among friends, families and caregivers is crucial. Promoting kindness is critical for happiness for all.
